# Androgen receptor transcriptionally regulates semaphorin 3C in a GATA2-dependent manner

**DOI:** 10.18632/oncotarget.14168

**Published:** 2016-12-25

**Authors:** Kevin J. Tam, Kush Dalal, Michael Hsing, Chi Wing Cheng, Shahram Khosravi, Parvin Yenki, Charan Tse, James W. Peacock, Aishwariya Sharma, Yan Ting Chiang, Yuzhuo Wang, Artem Cherkasov, Paul S. Rennie, Martin E. Gleave, Christopher J. Ong

**Affiliations:** ^1^ Vancouver Prostate Centre, Vancouver General Hospital, Vancouver, BC, Canada; ^2^ Department of Surgery, University of British Columbia, Vancouver, BC, Canada; ^3^ Department of Experimental Therapeutics, BC Cancer Agency, Vancouver, BC, Canada; ^4^ Department of Urologic Sciences, University of British Columbia, Vancouver, BC, Canada

**Keywords:** SEMA3C, androgen receptor, GATA2, FOXA1, POU2F1

## Abstract

The androgen receptor (AR) is a member of the nuclear receptor superfamily of transcription factors and is central to prostate cancer (PCa) progression. Ligand-activated AR engages androgen response elements (AREs) at androgen-responsive genes to drive the expression of gene batteries involved in cell proliferation and cell fate. Understanding the transcriptional targets of the AR has become critical in apprehending the mechanisms driving treatment-resistant stages of PCa. Although AR transcription regulation has been extensively studied, the signaling networks downstream of AR are incompletely described. Semaphorin 3C (SEMA3C) is a secreted signaling protein with roles in nervous system and cardiac development but can also drive cellular growth and invasive characteristics in multiple cancers including PCa. Despite numerous findings that implicate SEMA3C in cancer progression, regulatory mechanisms governing its expression remain largely unknown. Here we identify and characterize an androgen response element within the SEMA3C locus. Using the AR-positive LNCaP PCa cell line, we show that SEMA3C expression is driven by AR through this element and that AR-mediated expression of SEMA3C is dependent on the transcription factor GATA2. SEMA3C has been shown to promote cellular growth in certain cell types so implicit to our findings is the discovery of direct regulation of a growth factor by AR. We also show that FOXA1 is a negative regulator of SEMA3C. These findings identify SEMA3C as a novel target of AR, GATA2, and FOXA1 and expand our understanding of semaphorin signaling and cancer biology.

## INTRODUCTION

The androgen receptor (AR) is a 110 kDa member of the nuclear hormone receptor (NR) superfamily of transcription factors. Like other nuclear receptors, the AR is a modular protein which regulates gene expression in a ligand-dependent manner by engaging conserved DNA motifs and promoting transcription. Structurally, the AR consists of an N-terminal domain (NTD), a DNA-binding domain (DBD), a hinge region, and a C-terminal ligand-binding domain (LBD). The NTD and LBD domains contain AF1 and AF2 regions, respectively, that, together, serve to recruit ancillary proteins required for transcription initiation [1, 2]. The LBD contains an androgen-binding pocket that has been exploited therapeutically in the development of antiandrogen compounds for clinical application [3]. The DBD contains two zinc finger motifs which recognize and bind to androgen response elements (AREs) such as inverted repeats of AGAACA [2, 4–6]. Upon binding dihydrotestosterone (DHT), the AR translocates to the nucleus where it recruits co-activators, RNA polymerase II, and other components of the basal transcriptional complex at AREs found in the vicinity of androgen-regulated genes [7, 8]. AR is additionally known to cooperate with pioneering factors GATA2, FOXA1, and OCT1 to promote gene transcription through chromatin remodeling and DNA looping [9–13]. The AR holds important roles in normal prostate development and physiology, in the maintenance of secondary male characteristics, and in sexual function in the adult male; however, its dysregulation is also largely responsible for prostate cancer (PCa) development and progression [14–19]. Accordingly, AR is the focus of many translationally-driven investigational studies. PCa is the most commonly-occurring cancer among North American men and despite initially favourable response to treatment, progression to metastatic castration-resistant prostate cancer (CRPC) is frequent [20] for which treatments are palliative. Several lines of evidence support the notion that the AR is vital to early PCa but also to CRPC [21]. For example, AR transcriptional targets include genes related to cell proliferation and survival such as M phase cell cycle progression genes [22–24]. Furthermore, PCa tumours generally respond well to androgen blockade for at least a period of time. In addition, CRPC is often marked by: retention or amplification of the AR, biochemical recurrence of AR target genes such as prostate-specific antigen (PSA), and mutations to the AR rendering it constitutively active and refractory to the actions of antiandrogens [3]. Collectively, these observations suggest a causal role for AR in disease progression and underscore the importance of AR blockade and identification of novel AR targets whose expression by AR facilitate disease progression.

The semaphorin family of signaling proteins constitutes a large grouping of membrane-associated or secreted chemotactic factors that are involved in embryogenesis and neurogenesis. Semaphorins assist in neuronal outgrowth and axon guidance by establishing molecular gradients that guide cell movement [25–28]. Since their initial discovery, semaphorins have been implicated in a variety of different biological processes ranging from immunity to cell motility [29, 30]. Twenty different semaphorin family members have been identified in mammals and their roles in cancer development are becoming increasingly evident [31–34]. Semaphorin 3C (SEMA3C) is a secreted class 3 semaphorin and functions in endothelial, cardiac, and alveolar development [35–38]. SEMA3C is also involved in lung, gastric, ovarian, and breast cancer development and is a driver of glioma stem cell tumourigenicity [39–43]. Recent reports have implicated SEMA3C in angiogenesis [44, 45] and have also highlighted the significance and clinical relevance of SEMA3C in prostate cancer [46–48]. Additionally, SEMA3C and its receptors are frequently mutated and upregulated in PCa [32, 49–51]. Despite ongoing improvements to our understanding of the normal and pathological roles of semaphorins, reports describing their regulation are exceedingly scarce. In a genome-wide ChIP-Seq study that identified androgen receptor binding sites (ARBSs) across the genome of prostate cancer cell lines, several putative ARBSs in the SEMA3C genomic and upstream sequence were identified [52].

Given the implication of both AR and SEMA3C in prostate cancer development, in combination with the discovery of ARBSs within the *SEMA3C* locus, we hypothesized that *SEMA3C* is a transcriptional target of the androgen receptor. The concept that some semaphorins are hormone-regulated is not unprecedented and it has been reported that SEMA3G is upregulated by R1881 in prostate cells [16] and that SEMA3B and SEMA3F are regulated by estrogens in ovarian cancer cells [53]. In the present study, we provide new evidence that in AR-positive LNCaP cells, SEMA3C is upregulated by androgen-stimulated AR. We also assign function to an ARE discovered at one of the putative ARBSs identified by Yu *et al* using gel-shift, chromatin immunoprecipitation, and reporter gene assays. Collectively these data demonstrate that SEMA3C is a direct transcriptional target of AR. Lastly, we show that GATA2 is a necessary coactivator of AR-mediated expression of SEMA3C and that FOXA1 is a negative regulator of SEMA3C. These results reveal several previously unrecognized mechanisms of SEMA3C regulation. Furthermore, considering SEMA3C has been shown to promote growth in certain cell types [35, 54], this work also distinctly shows the regulation of a growth factor by the androgen receptor.

## RESULTS

### ARE and GATA2 DNA motifs at the human SEMA3C locus

**Figure 1:**
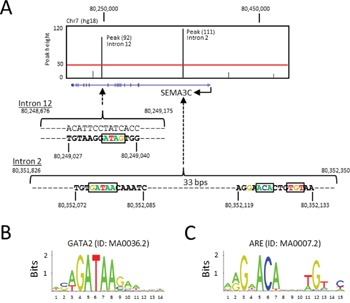
ARE and GATA2 DNA motifs at the human SEMA3C locus **A**. The ChIP-Seq peaks (vertical black bars) from Yu *et al* were overlaid on the SEMA3C locus (blue horizontal line: exons are shown as vertical blue bars) in the UCSC Genome Browser (hg18). A red horizontal line illustrates the average peak height from the ChIP-Seq experiment. The DNA sequences containing the ARE and GATA2 motifs, as predicted by the Patser program, are shown within the two peaks that span from 80,351,826 to 80,352,350 in intron 2 and from 80,248,676 to 80,249,175 in intron 12, respectively. **B**. A consensus GATA2 motif documented in the JASPAR database is illustrated as a sequence logo. **C**. A consensus ARE motif documented in the JASPAR database is illustrated as a sequence logo.

The ChIP-Seq study from Yu *et al* [52] reported a total of 44,536 genomic regions (or peaks) bound by the AR protein (ARBSs) in LNCaP cells under treatment with R1881. The average peak height, an indication of the amount of DNA bound by the AR, is 30 units. The annotation of the ChIP-Seq data by the CompleteMOTIFs program has identified 8 peaks that are within 500 kb of the transcription start site (TSS) of the *SEMA3C* gene, five of which are shown in Figure 1A in addition to three other peaks upstream of SEMA3C (not shown) which have heights lower than the average value. As illustrated in Figure 1A, two ChIP-Seq peaks have heights of 111 and 92, both of which are significantly higher than the average value and are in the 95^th^ percentile; the coding sequences are located on the reverse strand of chromosome 7 (hg18). The two ARBSs span 525 and 500 basepairs (bps) and are located in intron 2 (34.5 kb downstream of TSS) and intron 12 (137.7 kb downstream of TSS) of the *SEMA3C* gene, respectively. The DNA sequences at the ARBSs were scanned for the presence of ARE and GATA2 motifs, using a motif scanning program, Patser. An ARE DNA motif was found within the ARBS peak at intron 2 (*p* = 6.46 × 10^-6^), while no ARE motif was found within the peak at intron 12. Prospective GATA2 motifs were identified in both intron 2 (*p* = 1.02 × 10^-4^) and intron 12 (*p* = 1.17 × 10^-4^) which resemble the GATA2 motif as defined by JASPAR motif database (Figure 1B). We began our investigation of AR-mediated regulation of *SEMA3C* by examining the putative ARE that spans from 80,352,119 to 80,352,133 in the intron 2 of SEMA3C, which shares strong resemblance to the ARE motif (Figure 1C) as defined in the JASPAR motif database [2, 4–6, 55].

### *SEMA3C* is an androgen receptor-regulated gene

**Figure 2:**
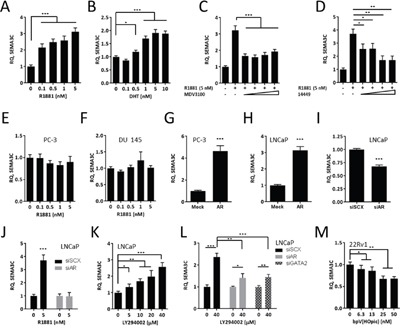
SEMA3C is an androgen receptor-regulated gene LNCaP were treated with increasing concentrations (0 to 5 nM) of the synthetic androgen, R1881 **A**., and increasing concentrations (0 to 10 nM) of dihydrotestosterone (DHT; **B**.) followed by detection of SEMA3C mRNA levels by qPCR; relative quantities (RQ) are presented. R1881-stimulated LNCaP cells were treated with increasing concentrations (6.25, 12.5, 25, and 50 μM) of Enzalutamide (MDV3100; **C**.) or of the AR DBD inhibitor VPC-14449 (14449; **D**.) followed by SEMA3C mRNA level detection by qPCR. AR-negative PCa lines PC-3 and DU 145 did not upregulate SEMA3C in response to R1881 **E** & **F**. PC-3 and LNCaP cells were transfected with mock or AR overexpression plasmids followed by qPCR for SEMA3C **G** & **H**. LNCaP cells were transfected with siRNA directed against AR (siAR) or scrambled siRNA (siSCX) followed by qPCR for SEMA3C I. LNCaP cells were treated with R1881 (5 nM) in the presence of siAR followed by qPCR for SEMA3C **J**. LNCaP were treated with PI3K inhibitor LY294002 at the indicated concentrations or DMSO and monitored for SEMA3C message expression **K**. LNCaP were treated with LY294002 (40 μM) in the presence of siRNA for AR (siAR) or GATA2 (siGATA2) followed by qPCR for SEMA3C **L**. 22Rv1 were treated with PTEN inhibitor bpV(HOpic) at the indicated concentrations or DMSO and monitored for SEMA3C message expression **M**. Data represent mean, ± SD; * *p* < 0.05, ** *p* < 0.01, *** *p* < 0.001.

Considering that *SEMA3C* contains an ARE in its second intron, *SEMA3C* may be an androgen-regulated gene; androgen receptor is known to act through distantly-located (including intronic) ARBSs through DNA looping [11, 13, 18, 56]. To first assess the androgen-responsiveness of *SEMA3C*, AR-positive LNCaP cells were treated with the synthetic androgen R1881 or naturally-occurring ligand, DHT, and tested for SEMA3C expression. Consistent with our hypothesis, SEMA3C mRNA levels increased in a dose-dependent manner upon treatment with both R1881 and DHT (Figure 2A and 2B, respectively). R1881 triggered increases in SEMA3C mRNA levels from 2.2 to 3.1 over that of vehicle control while DHT reached a maximum induction of 1.9-fold over vehicle control at 5 nM. These results are supported by existing microarray datasets [57] which examine the gene expression profiles of LNCaP in response to R1881 over time. Data mining [58] of these datasets (GEO accession number GDS2034) showed that SEMA3C mRNA levels increased in a time-dependent manner (Supplementary Figure 1). To test if antiandrogens could influence SEMA3C expression, we administered increasing concentrations of MDV3100 (Enzalutamide), an androgen competitor that competes with androgens for the AR LBD [59], to R1881-stimulated LNCaP cells. MDV3100 inhibited SEMA3C expression by over 50% at all concentrations of MDV3100 examined (Figure 2C). A recently-developed small molecule AR inhibitor (“VPC-14449”) with well-characterized AR DBD-interfering activity [60, 61] was also capable of inhibiting R1881-mediated induction of SEMA3C expression (Figure 2D). VPC-14449 inhibited R1881-induced expression of SEMA3C by 43%, 42%, 73%, and 73% at 6.25, 12.5, 25, and 50 μM, respectively. These findings are consistent with data mining of a previously published microarray dataset on MDV3100- and VPC-14449-treated R1881-stimulated LNCaP cells [61] where it was shown that administration of MDV3100 and VPC-14449 decreased SEMA3C expression by 20% (*p* = 0.10) and 25% (*p* = 0.04), respectively. To reinforce the idea that SEMA3C expression requires AR, we also showed that SEMA3C levels were not induced by R1881 in the AR-negative prostate cancer cells lines, PC-3 and DU 145, over the same concentrations (Figure 2E & 2F). Additionally, ectopic expression of AR in PC-3 and LNCaP led to a 4.6 and 3.1-fold increase in SEMA3C expression, respectively (Figure 2G & 2H). In the reciprocal experiment, knockdown of AR in LNCaP resulted in a 32% reduction in SEMA3C expression (Figure 2I); verification of AR knockdown was confirmed by Western blot analysis (Supplementary Figure 2A). To ascertain whether R1881-induced SEMA3C expression was by way of AR, we treated LNCaP cells with R1881 in the presence and absence of small interfering RNA (siRNA) for AR (siAR). These experiments showed that R1881-induced expression of SEMA3C is AR-dependent (Figure 2J); knockdown of AR in these studies was confirmed by Western blot analysis (Supplementary Figure 2B). Collectively, these results demonstrate that steroid-activated AR can trigger upregulation of SEMA3C.

The PI3K pathway is frequently mutated in prostate cancer and *PTEN* is mutated in up to 40% of advanced prostate cancer patients. The inhibition of PI3K signaling was shown to upregulate AR-regulated genes [62] therefore we asked whether inhibiting PI3K using LY294002 could trigger upregulation of SEMA3C. In accordance with findings shown by Carver *et al*, inhibiting PI3K using LY294002 caused upregulation of SEMA3C mRNA (Figure 2K). Furthermore, induction of SEMA3C expression by LY294002 was significantly diminished when either AR or GATA2, a protein that cooperates with AR, were knocked down (Figure 2L); knockdown of AR and GATA2 was confirmed by Western blot analysis (Supplementary Figure 2C). Conversely, inhibiting PTEN using bpV(HOpic) in the PTEN-positive PCa line, 22Rv1, caused a downregulation of SEMA3C (Figure 2M). Phospho-Akt was used to monitor the effects of LY294002 and bpV(HOpic) on PI3K activity (Supplementary Figure 2D & 2E).

### The androgen receptor associates with the SEMA3C intron 2 ARE

**Figure 3:**
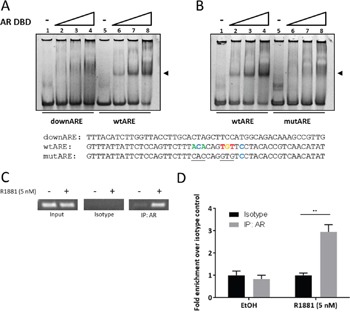
The androgen receptor associates with the SEMA3C intron 2 ARE In electrophoretic mobility shift assays, 50 basepair oligonucleotides at a final concentration of 1.875 μM were combined with increasing concentrations (0, 0.5, 1.0, 2.0 μM) of purified human androgen receptor DNA-binding domain (AR DBD) and run on a non-denaturing acrylamide gel. A shift (at arrow) was observed when AR DBD was combined with oligonucleotide containing the intron 2 ARE (wtARE) but not when combined with either a 50 bp oligonucleotide mapping to ∼200 bp downstream of the intron 2 ARE (downARE) **A**. nor when combined with an oligonucleotide with transversion mutations to six bases of the ARE (mutARE) **B**. Sequences of the oligonucleotides used for the assay are shown below; sequences shown are complementary to those of Figure 1: Bases matching the JASPAR motif are shown in colour; mutations are underlined. ChIP assays were carried out on lysates of LNCaP treated with 0.05% ethanol (vehicle control) or 5 nM R1881. PCR was performed on 1% input (Input), isotype-matched control (Isotype), and AR immunoprecipitates (IP: AR). **C**. End-point PCR showed abundant levels of SEMA3C ARE amplicon in input and undetectable levels in isotype control irrespective of R1881 treatment. Ethanol-treated AR immunoprecipitates showed low but detectable levels of SEMA3C ARE amplicon whereas R1881 triggered enriched SEMA3C ARE amplicon in AR immunoprecipitates. Results were confirmed by qPCR **D**.; values represent fold enrichment over isotype control of the same treatment condition. ± SD; ** *p* < 0.01.

We next set out to determine if the AR acts in *cis*- at the intron 2 ARE or if the observed SEMA3C induction is the result of upregulation of intermediary factors or pleotropic effects of an activated AR axis. We first tested the capacity of the AR to interact with the SEMA3C intron 2 ARE using an electrophoretic mobility shift assay utilizing purified human AR DNA-binding domain (AR DBD) and a 50 bp double-stranded oligonucleotide centred around the SEMA3C intron 2 ARE (wtARE). Whereas the wtARE oligonucleotide was shifted by the AR DBD, a 50 bp oligonucleotide mapping to an area roughly 200 bps downstream of the endogenous ARE (downARE) did not (Figure 3A, compare lanes 2-4 and 6-8). Moreover, incorporation of transversion mutations to six of the core nucleotides constituting the putative ARE (mutARE) abolished the gel-shift observed with the wtARE oligonucleotide (Figure 3B, compare lanes 2-4 and 6-8). Thus, the AR DBD is capable of interacting with this putative intragenic ARE.

In canonical NR signaling, NRs are recruited to response elements in the vicinity of target genes in a ligand-inducible manner. To determine if AR is recruited to the SEMA3C locus in this fashion, we used chromatin immunoprecipitation (ChIP) with an AR-specific antibody on lysates from EtOH or R1881-treated LNCaP cells and amplified a 150 bp region mapping to the SEMA3C intronic ARE. R1881 treatment of LNCaP cells resulted in the recruitment of AR to genomic SEMA3C ARE as shown by elevated levels of the SEMA3C ARE amplicon in both end-point (Figure 3C) and quantitative PCR (Figure 3D). In end-point PCR, inputs contained detectable levels of SEMA3C intronic ARE amplicon but no amplicon was detected in immunoprecipitation with isotype control (Figure 3C). GAPDH amplicon was not enriched for by AR immunoprecipitation with R1881 treatment nor was any detectable in the isotype control samples (data not shown). In quantitative PCR, a 3-fold increase in enrichment over isotype control was observed in the R1881-treated samples whereas no enrichment was observed in the EtOH treatment (Figure 3D). Since AR occupancy is a strong indicator of AR-regulation [16], these assays support the notion that SEMA3C is an androgen receptor-regulated gene.

### Androgen receptor transactivates the SEMA3C intron 2 ARE

**Figure 4:**
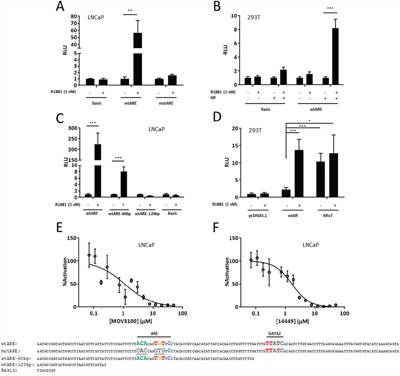
Androgen receptor transactivates the SEMA3C intron 2 ARE **A**. LNCaP cells were transfected with empty pGL3-Basic reporter plasmid (Basic), SEMA3C intron 2 ARE reporter plasmids (wtARE), or reporter plasmids where the ARE was mutated (mutARE). Sequences cloned into reporter plasmids are shown below; sequences shown are complementary to those of Figure 1: Bases matching the JASPAR motif are shown in colour and ARE and GATA2 elements are indicated; mutations are underlined. Cells were then treated with R1881 at 5 nM or vehicle control (0.05% EtOH) and harvested for measurement of relative luminescence (RLU). **B**. 293T were co-transfected with reporter plasmids (Basic or wtARE) and AR overexpression plasmids followed by treatment with R1881 at 1 nM or vehicle. **C**. LNCaP cells were transfected with wtARE, wtARE with the final 60 basepairs truncated (wtARE-60bp), wtARE with the final 120 basepair truncated (wtARE-120bp), and Basic (sequences are shown below). The wtARE construct contains both the AR and GATA2 motif; the wtARE-60bp construct has the GATA2 motif removed but retains the AR motif; the wtARE-120bp construct contains neither the AR nor the GATA2 motif. Transfected cells were then treated with R1881 at 5 nM or vehicle. **D**. 293T cells were transfected with wtARE and either wtAR or ARv7 overexpression plasmid and subsequently treated with R1881 to 1 nM. Transfection with pcDNA3.1 served as a control. Values represent a fold increase over EtOH control (A-C) or fold increase over pcDNA3.1/EtOH treatment (D). Data represent mean, ± SD; * *p* < 0.05, ** *p* < 0.01, *** *p* < 0.001. LNCaP were transfected with wtARE and co-treated with R1881 (0.1 nM) and dosages of MDV3100 **E**. or VPC-14449 **F**. ranging from 0.04 to 50 μM. MDV3100 IC_50_ = 1.1 μM and 14449 IC_50_ = 1.7 μM. ± SEM. For all luciferase assays, renilla luciferase was used to normalize readings.

AR-mediated gene transcription culminates in the recruitment of RNA polymerase II, coactivators, and other members of the pre-initiation complex to the promoter region of androgen-responsive genes. This assembly is largely coordinated through AR's AF1, AF2, and BF3 domains [7, 63–65]. To determine if the isolated SEMA3C ARE is a platform capable of orchestrating these events, we utilized pGL3 reporter constructs bearing a one hundred and fifty bp region surrounding the SEMA3C intron 2 ARE placed upstream of the luciferase gene. Luciferase activity was increased 57 times by R1881 in LNCaP cells transfected with the SEMA3C ARE luciferase reporter construct (wtARE) but not in LNCaP transfected with the empty pGL3-Basic vector (Basic; Figure 4A). Luciferase activity was not induced by R1881 in LNCaP transfected with a reporter construct bearing the 6 bp mutant form of the ARE that was described in Figure 3 (mutARE; Figure 4A). Similar results were obtained when 293T cells were co-transfected with reporter constructs and AR overexpression plasmids. R1881 induced an 8.2-fold increase in luciferase activity in 293T co-transfected with wtARE and AR, however, in the absence of co-transfection with AR, 293T cells showed only minor R1881-responsiveness (1.6-fold induction; Figure 4B). Minor R1881 induction of luciferase activity was observed in 293T cells transfected with Basic and AR (2.2-fold induction; Figure 4B) owing likely to cryptic regulatory elements in the empty reporter plasmid. No induction was observed in 293T cells transfected with Basic in the absence of AR co-transfection. In search of additional elements within our insert that are responsible for luciferase induction, a series of constructs with progressive 3′ deletions to wtARE insert were generated. Truncation of 60 bp from the full-length insert (wtARE-60bp) resulted in a drastic reduction in R1881 induction of luciferase activity (from 220 to 8.1-fold induction; Figure 4C) presumably due to the removal of one or more elements that support AR transcription initiation (discussed further below). The deletion of an additional 60 bp (wtARE-120bp), which removes the putative ARE, completely abrogated R1881 induction of luciferase activity (from 8.1-fold to no induction; Figure 4C). In PCa, following castration, a restored AR axis is thought to be one of the mechanisms that precipitate the onset of castrate-resistant disease. Restoration of AR activity despite castrate levels of androgens is thought to be through mechanisms that include overexpression of, or mutations to, the AR. The constitutively active AR splice variant, ARv7, which lacks the ligand-binding domain, could represent one such example. Co-transfection of the ARv7 overexpression plasmid and the wtARE reporter plasmid into 293T cells resulted in luciferase activity comparable to that of ligand-activated wild-type AR (Figure 4D) which was irrespective of R1881 treatment. Collectively, these results demonstrate that the identified ARE can coordinate the recruitment of factors necessary for AR-mediated transcription initiation. For biochemical comparison, the luciferase transcriptional output from the SEMA3C intron 2 ARE was titrated against concentrations of MDV3100 and VPC-14449 in R1881-activated LNCaP cells. In these studies, the IC_50_ of MDV3100 and VPC-14449 was discovered to be 1.1 μM and 1.7 μM, respectively (Figure 4E & 4F). This dose-dependent AR inhibition by MDV3100 and VPC-14449 is consistent with the transcriptional inhibition observed for other ARE-bearing reporter constructs in LNCaP and other cell lines [60, 61].

### R1881-induction of SEMA3C expression is GATA2-dependent

**Figure 5:**
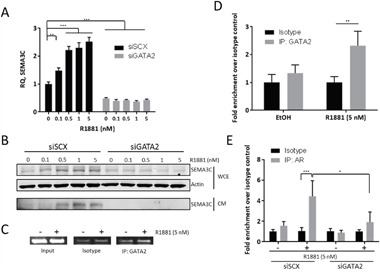
R1881-induction of SEMA3C expression is GATA2-dependent We examined R1881-induced expression of SEMA3C in the absence of GATA2 to confirm findings from previous microarray studies showing that knockdown of GATA2 decreases SEMA3C expression. When compared to LNCaP treated with scrambled siRNA (siSCX), knockdown of GATA2 (siGATA2) triggered a significant decrease in basal SEMA3C expression and completely attenuated R1881-mediated dose-dependent induction of SEMA3C as shown by qPCR **A**. These observations were confirmed at the protein level by Western blot analysis **B**. of both conditioned media (CM) and whole cell extract (WCE) where total actin served as loading control. In chromatin immunoprecipitation assays, SEMA3C ARE amplicon was shown be enriched in GATA2 immunoprecipitates of lysates from LNCaP cells treated with R1881 as shown by end-point **C**. and qPCR **D**. indicating an R1881-dependent recruitment of GATA2 to the SEMA3C intron 2 ARE. Input = 1% input, Isotype = isotype-matched control antibody, IP: GATA2 = GATA2 immunoprecipitates. PCR primers for these experiments were the same as those for Figure 3 and map to the SEMA3C intron 2 ARE. ChIP qPCR values represent a fold enrichment over isotype control of the same treatment condition. Chromatin immunoprecipitation assays previously showing R1881-induced recruitment of AR to the SEMA3C ARE were repeated in the presence of siRNA to GATA2 **E**. Values represent a fold enrichment over isotype control of the same treatment condition. Data represent mean, ± SD; *p < 0.05, ** *p* < 0.01, *** *p* < 0.001.

The GATA family of transcription factors are pioneering factors that control gene expression through epigenetic chromatin remodeling [66, 67]. One such member, GATA2, has been shown to cooperate with the AR in the regulation of androgen-dependent genes [11, 68]. GATA2 has roles in development and has recently been ascribed various roles in prostate cancer development [9, 10, 69, 70]. Related family member, GATA6, is known to regulate SEMA3C in cardiac neural crest [71]. Inspection of the DNA sequence near the SEMA3C intron 2 ARE using Patser software revealed a GATA2 consensus sequence at the genomic coordinates 80,352,072 to 80,352,085 situated thirty-three bps downstream of the ARE (Figure 1A & 1B). In reporter gene assays, truncation of this element from the full-length reporter construct corresponded to a nearly 30-fold decrease in R1881-inducibility (Figure 4C). In addition to this, in microarray studies we found that GATA2 silencing led to a five-fold decrease in SEMA3C expression in LNCaP (FDR<0.001; NCBI, Gene Expression Omnibus, GEO accession number GSE49342). This data was validated by qPCR where silencing of GATA2 decreased basal levels of SEMA3C by 50% compared to LNCaP treated with negative control scrambled siRNA (Figure 5A). Furthermore, knockdown of GATA2 also prevented R1881-induced expression of SEMA3C which was reflected at both the mRNA and protein levels (Figure 5A & 5B). These findings are in agreement with reports describing a dependency by androgen-regulated genes on GATA2 [10, 11, 68]. Knockdown of GATA2 was verified by Western blot analysis (Supplementary Figure 3A). We employed ChIP to investigate whether GATA2 is also recruited to the intron 2 ARE region in an androgen-dependent manner. Similar to ChIP results seen with AR (Figure 3C & 3D), GATA2 was recruited to the SEMA3C intron 2 ARE region in an R1881-dependent manner shown by both end-point PCR (Figure 5C) and by qPCR (Figure 5D) implying that GATA2 is implicated in AR-driven expression of SEMA3C. Quantitative PCR revealed a 2.3-fold enrichment of GATA2 to the intron 2 ARE region upon R1881 treatment whereas no significant enrichment was seen in the EtOH treatment. Silencing of GATA2 also abrogated R1881-induced recruitment of AR to the ARE in ChIP (4.4- versus 1.9-fold enrichment in scrambled versus GATA2 knockdown, respectively; Figure 5E). Knockdown of GATA2 was confirmed by Western blot (Supplementary Figure 3B). The presence of a GATA2 motif in such close proximity to the ARE, the dependency on GATA2 in androgen-induced expression of SEMA3C, in reporter gene assays, and in recruitment of AR to the ARE, and the co-recruitment of AR and GATA2 to the intron 2 ARE, together strongly suggest a coordinated effort between AR and GATA2 in the regulation of SEMA3C.

### FOXA1 negatively regulates SEMA3C expression

**Figure 6:**
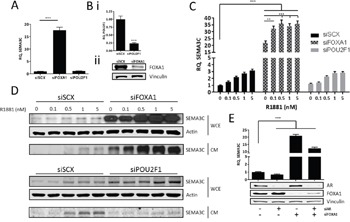
FOXA1 negatively regulates SEMA3C expression We assessed the effect of silencing of FOXA1 and POU2F1 on SEMA3C message levels. **A**. Knockdown of FOXA1 (siFOXA1) triggered an increase in SEMA3C levels when compared to cells treated with scrambled siRNA (siSCX); knockdown of POU2F1 (siPOU2F1) had no effect on SEMA3C expression. Knockdown of these genes was confirmed by qPCR (POU2F1) or Western blot (FOXA1; **B**.). In siFOXA1-treated cells where SEMA3C levels were already elevated, SEMA3C expression was further increased upon R1881 stimulation shown at both the message **C**. and protein **D**. level. Knockdown of POU2F1 had little effect on R1881 induction of SEMA3C (C, D). WCE: whole cell extract; CM: conditioned media. **E**. LNCaP cells were knocked down with siAR, siFOXA1, or both siAR and siFOXA1 and monitored for SEMA3C expression. Compared to LNCaP treated with scrambled siRNA, siAR-treated cells had decreased SEMA3C expression while siFOXA1 and siAR+siFOXA1-treated cells had elevated SEMA3C expression. Knockdown of AR and FOXA1 was confirmed by Western blot analysis (E). Data represent mean, ± SD; ** *p* < 0.01, *** *p* < 0.001.

The forkhead box (FOX) and POU-homeodomain family of transcription factors have well-established roles in the expression of genes necessary for development [72, 73]. Importantly, FOXA1 and POU2F1 (also known as OCT1) are also known to cooperate with GATA2 and AR in the expression of androgen-regulated genes [11, 12, 74] and FOXA1 is frequently mutated in advanced prostate cancer patients [75–77]. This prompted us to explore whether FOXA1 and POU2F1 are also involved in SEMA3C expression. To this end, we examined whether SEMA3C expression was altered following FOXA1 and POU2F1 knockdown. Knockdown of FOXA1 and POU2F1 resulted in an 18 and 1.2-fold induction of SEMA3C expression, respectively, over scrambled siRNA transfection control, strongly indicating that FOXA1 negatively regulates SEMA3C whereas POU2F1 seems not to play a role in SEMA3C expression (Figure 6A). Knockdown of POU2F1 and FOXA1 was assessed by qPCR and Western blot, respectively (Figure 6Bi & 6Bii). Whereas FOXA1 was detectable by Western blot, POU2F1 was not, therefore POU2F1 knockdown was monitored by qPCR. To determine if FOXA1 or POU2F1 has an impact on R1881-induced SEMA3C expression, LNCaP were treated with R1881 in combination with FOXA1 or POU2F1 knockdown. In the absence of FOXA1, basal SEMA3C levels increased substantially and further increased upon treatment with R1881 at both the message and protein level (Figure 6C & 6D). SEMA3C mRNA levels increased between 47% and 64% with R1881 treatments over vehicle control in the absence of FOXA1 (Figure 6C). R1881 induction of SEMA3C in the absence of POU2F1 did not differ significantly from that seen in cells treated with scrambled siRNA control (Figure 6C & 6D). To clarify if FOXA1 silencing-mediated induction of SEMA3C was dependent on AR, we silenced AR and FOXA1 simultaneously and found that even in the absence of AR, knockdown of FOXA1 triggered induction of SEMA3C, albeit less than in the presence of AR (21 versus 12-fold induction of SEMA3C in siFOXA1 and siFOXA1+siAR, respectively), indicating that the observed induction is not dependent on AR (Figure 6E). Efficacy of knockdown of AR and FOXA1 was confirmed by Western blot (Figure 6E). Collectively these results suggest that while POU2F1 seems not to be involved in SEMA3C regulation, FOXA1 is a negative regulator of SEMA3C and that this suppression is independent of AR. In light of a propensity for FOXA1 mutations in advanced PCa, aberrant FOXA1 signaling may contribute to elevated SEMA3C expression. The effect of knockdown of each of GATA2, FOXA1, and POU2F1 on SEMA3C expression was repeated in C4-2 cells and results were concordant with those seen in knockdown of the same genes in LNCaP (Supplementary Figure 4A). Knockdown of GATA2 resulted in an 87% reduction of SEMA3C levels while knockdown of FOXA1 and POU2F1 triggered an 8.3 and 1.5-fold induction of SEMA3C, respectively. Knockdown was verified by either Western blot or qPCR (Supplementary Figure 4B).

## DISCUSSION

A more complete understanding of the set of androgen receptor-regulated genes that drive growth and survival will be instrumental to the development of more efficacious prostate cancer therapies and will be of particular importance in curtailing progression to castration-resistant stages of the disease. The ARE in the second intron of SEMA3C identified based on initial studies by Yu *et al*, is located roughly 30 kb downstream of the TSS for this gene. In many AR gene targets, the corresponding AREs are situated downstream of the TSS at distances of tens of kilobases [11, 16]. Here we demonstrate that SEMA3C expression is regulated by the androgen receptor in a prototypical way and that androgens induce SEMA3C expression, that the AR is recruited to the *SEMA3C* locus in an androgen-dependent fashion, and that the intronic ARE can bind to and be transactivated by the AR. We further demonstrate that GATA2 is critical to this process and that FOXA1 is a negative regulator of SEMA3C expression. FOXA1 suppression of SEMA3C expression, although additive in nature with those of AR, is not dependent on AR.

Although pathways downstream of AR are known to include those that are involved in cell cycle progression and cell fate [15, 78–80], our work shows that AR *directly* regulates the expression of a growth factor and may therefore have profound implications. Considering the co-occurrence of aberrant AR and SEMA3C in advanced prostate cancer, we speculate that restored AR signaling in advanced PCa drives SEMA3C expression which in turn propels PCa progression. SEMA3C is known to have roles in embryogenesis, therefore, its upregulation might also confer stem-like phenotypes to cancer cells and contribute to tumor heterogeneity frequently observed in tumor histopathology of PCa patients. It is also worth noting that SEMA3C is associated with tumor cell motility [35, 42, 47, 54] and may therefore contribute to cancer cell dissemination. This propensity for motility may be especially important in CRPC where elevated SEMA3C expression may confer cancer cells with invasive phenotypes that contribute to metastasis. Whether SEMA3C expression drives CRPC progression or simply accompanies restored AR activity remains to be seen and will require SEMA3C gain-of-function and loss-of-function studies. Remarkably, the regulatory networks governing semaphorin expression are largely unknown despite the fact that SEMA3C and its kin are heavily implicated in cancer. SOX4, GATA-6, and Twist1 are three transcription factors with reported involvement in the regulation of SEMA3C [71, 81–83] but to our knowledge, hormone regulation of SEMA3C has not yet been reported. Our success in characterizing the *SEMA3C* gene in this way underscores the need for a more comprehensive understanding of the biological roles of SEMA3C in the development of CRPC.

The co-recruitment of AR and GATA2 to a region well downstream of the transcriptional start site of SEMA3C (Figure 3D & 5D) would imply involvement of a DNA looping mechanism whereby the ARE and SEMA3C proximal promoter are brought together through a bridge consisting of AR and GATA2 and likely other proteins. AR and GATA2 are known to associate with distally-located ARBS as a form of gene regulation of AR-regulated genes [10, 11, 13]. Techniques such as ChIP-on-chip, ChIP-Seq, or ChIP Combined with Chromosome Conformation Capture (5C) will be required to prove that the intron 2 ARE is cooperating with AR and GATA2 via DNA looping. Despite the known role of GATA2 in chromatin remodelling, our reporter gene assays further suggest that GATA2 also directly supports AR transcription initiation since deletion of the GATA2 motif drastically diminishes R1881-mediated induction of luciferase activity (Figure 4C). Nevertheless, the notion that GATA2 is a driver of PCa is seemingly consistent with our findings that GATA2 promotes the expression of oncogenic SEMA3C. FOXA1's inhibitory effect on SEMA3C expression may also be of significance given that FOXA1, which is often found to be mutated in advanced PCa, may account for elevated SEMA3C expression in advanced prostate cancer.

In addition to an ARBS in intron 2, Yu and colleagues also identified an ARBS in intron 12 of SEMA3C [52]. Inspection of the ARBS in intron 12 using Patser software did not reveal consensus sequences for the androgen receptor (data not shown) but presumably AR associates with this region of DNA through intermediary transcription factors or coregulators. Patser software analysis did, however, reveal a GATA2 consensus sequence in intron 12 of SEMA3C spanning the genomic coordinates 80,249,027 to 80,249,040 on the reverse strand (Figure 1A). It is conceivable that recruitment of AR to this genomic region is GATA2-dependent, further implicating GATA2 in AR-mediated regulation of SEMA3C. The functional significance of the GATA2 element identified in intron 12 and whether the intron 12 ARBS and GATA2 element are acting in concert with the *cis*-acting elements of intron 2 remain to be determined. Our results also raise the possibility that other semaphorins or their receptors fall under AR, GATA2, or FOXA1 regulation, especially given that many semaphorin family members are implicated in cancer. Indeed, Yu *et al*'s results also identify ARBSs in nearly all other class 3 semaphorins, most notably three ARBSs in another class 3 semaphorin with well-documented tumor-promoting activity, SEMA3E. Conversely, receptors to semaphorins have been shown to promote AR activity [84].

Our findings identify SEMA3C as a novel target of the androgen receptor and further show that GATA2 is indispensable to AR-mediated expression of SEMA3C. AR propels castration-resistant forms of PCa, therefore, identification of genes downstream of AR which mediate disease progression may unveil therapeutic targets for castrate and enzalutamide-resistant forms of the disease. Accordingly, the identification of SEMA3C as a direct transcriptional target of AR offers SEMA3C as a target potentially available for therapeutic exploitation which would be independent of shortfalls associated in targeting AR itself. SEMA3C is an attractive target in this regard because SEMA3C's roles are diminished in adults. Furthermore, since SEMA3C is a secreted protein, the biological fraction of SEMA3C that is accessible for targeting by pharmacological agents is high.

## MATERIALS AND METHODS

### Bioinformatics and data set analysis

Previous ChIP-Seq data from Yu *et al* [52] was extracted from the NCBI Gene Expression Omnibus (GEO) database [85]. In particular, the file (‘GSM353644_jy10s123.allregions.txt.gz’), which contained enriched DNA regions (i.e. peaks) bound by the AR protein in LNCaP cells treated with R1881 (GEO sample accession: GSM353644), was parsed to a bedGraph format and visualized in the UCSC Genome Browser [86] to identify AR binding sites (ARBS) nearby to the SEMA3C (RefSeq accession number NM_006379) locus on chromosome 7 of the human reference genome (version: hg18). The file contains a total of 44,536 different AR binding sites across the human genome, and each region was annotated for the distance to nearby genes using a ChIP-Seq analysis program, CompleteMOTIFs [87]. The actual DNA sequences that compose each AR binding peak region (∼ 500 bps) at the SEMA3C locus were extracted and scanned for any presence of the ARE motif (15 bps) and GATA2 motif (14 bps), using a DNA motif scanning program, Patser [88]. The DNA frequency matrices that define the ARE motif (ID: MA0007.2) and GATA2 motif (ID: MA0036.2) was obtained from the JASPAR database [55].

### DNA sequences

Fifty basepair oligonucleotides centred around the SEMA3C intron 2 ARE were used for electrophoretic mobility shift assay. Oligonucleotides were purchased from Integrated DNA Technologies and annealed by ramping from 90°C to 25°C at 0.1°C per second; sequences are displayed in Figure 3. In reporter gene assays, 150 bases of genomic sequence bearing the ARE were cloned into the luciferase reporter backbone pGL3-Basic to generate the wtARE construct. Six of the basepairs constituting the putative ARE in wtARE were mutated by transversion mutations to generate the mutARE construct. Truncation mutants were generated by progressive 60 basepair deletions to the 3′ end of wtARE insert. Sequences are displayed in Figure 4. DNA sequences were cloned in the same reading orientation relative to luciferase as they were found relative to *SEMA3C* in the genome. Ectopic expression of AR or ARv7 was achieved by transient transfection of PC-3, LNCaP (Figure 2) or 293T (Figure 4) with overexpression vectors where wild type AR or ARv7 is placed under the control of the CMV promoter in the pcDNA3.1 vector (Invitrogen). Empty pcDNA3.1 vector served as a negative control. Plasmids were transfected using Lipofectamine 2000 (Invitrogen, Cat. No. 111668-027) for 24 hours.

### Cell culture

LNCaP (ATCC, CRL-1740), 22Rv1 (ATCC, CRL-2505), and C4-2 cells (kindly provided by Dr. Leland W.K. Chung, MD Anderson Cancer Center, Houston, TX) were cultured in RPMI 1640 supplemented with 10% FBS; PC-3 (ATCC, CRL-1435), DU 145 cells (ATCC, HTB-81), and HEK/293T cells (ATCC, CRL-11268) were cultured in 10% FBS DMEM. Cells were treated at the indicated concentrations of androgen or 0.05% ethanol as a vehicle control in 0.2% charcoal-stripped serum (CSS) in Opti-MEM (Gibco, Cat. No. 11058-021) for 24 hours for qPCR or 48 hours for Western blot unless otherwise stated. Cells were starved for 24 hours in 0.2% CSS in Opti-MEM prior to treatment with R1881 (Perkin-Elmer, Cat. No. NLP005) or DHT (Sigma-Aldrich, Cat. No. D-073). For inhibition studies involving Enzalutamide (MDV3100) or AR DBD inhibitor VPC-14449, LNCaP were co-treated with R1881 and MDV3100, VPC-14449, or DMSO as a vehicle control in 0.2% CSS in Opti-MEM. For LY294002 (EMD /Millipore, Cat. No. 440202) and bpV(HOpic) (Santa Cruz Biotechnology, Cat. No. sc-221377) treatment studies, cells were treated at the indicated concentrations of inhibitor or vehicle control (DMSO) overnight in serum- and phenol red-free RPMI 1640.

### Quantitative polymerase chain reaction

Messenger RNA levels of SEMA3C were measured by qPCR. Total RNA was extracted using TRIzol (Invitrogen, Cat. No. 15596018) and 2 μg of RNA was reverse-transcribed using random hexamers (Roche, Cat. No. R15504) and Superscript II (Invitrogen, Cat. No.18064-014). qPCR was carried out using a ΔΔCt method on an AB ViiA7 real-time PCR machine; reactions were prepared using Platinum SYBR Green (Invitrogen, Cat. No.11744-500) and GAPDH or actin served as an endogenous control. GAPDH primer sequences: 5′- caccagggctgcttttaactc (forward), 5′- gacaagcttcccgttctcag (reverse); actin primer sequences: 5′- gctcttttccagccttcctt (forward), 5′- cggatgtcaacgtcacactt (reverse); SEMA3C primer sequences: 5′- gacaatttgcgtgttggttg (forward), 5′- cggtcctgatcttcatcca (reverse); POU2FA primer sequences: 5′- atgaacaatccgtcagaaaccag (forward), 5′- gatggagatgtccaaggaaagc (reverse).

### Electrophoretic mobility shift assay (EMSA)

Electrophoretic mobility shift assay with the androgen receptor DBD was carried out as described elsewhere [61]. Briefly, complementary 50 basepair oligonucleotides centred around the SEMA3C intron 2 ARE were synthesized (Integrated DNA Technologies), annealed, and combined with purified AR DBD. Oligonucleotide at a final concentration of 1.875 μM was mixed with AR DBD at final concentrations of 0.5, 1.0, and 2.0 μM and incubated on ice for 30 minutes in loading buffer. Oligonucleotide alone and oligonucleotide-AR DBD mixtures were run on a 5% native polyacrylamide gel at 125 volts in 1X TBE at 4°C and visualized using SYBR Safe DNA Gel Stain (Invitrogen Cat. No. S33102). See Figure 3 for oligonucleotide sequences.

### Chromatin immunoprecipitation assay (ChIP)

2.5×10^6^ LNCaP cells were treated with 0.05% ethanol or 5 nM R1881 overnight and fixed in 1% formaldehyde for chromatin immunoprecipitation using the Millipore EZ-ChIP Chromatin Immunoprecipitation Kit protocol (Cat. No. 17-371). For end-point PCR, 2 μl of purified DNA was used for thermocycling: initial denaturing at 94°C for 3 minutes, followed by 33 cycles of 20 seconds 94°C denaturing, 30 seconds 52°C annealing, 30 seconds 72°C extension, and a single final 72°C 2 minute extension step. Products were run at 90 volts on a 2% agarose TBE gel and stained with SYBR Safe DNA Gel Stain. For qPCR, 1.5 μl of purified DNA was used per reaction. For ChIP qPCR reactions, SEMA3C intron 2 ARE primer sequences: 5′- aaatgccggtactggcctta (forward), 5′- gcttaaaggtcacaagattg (reverse); PCR primers amplify a 150 bp genomic region containing the SEMA3C intron 2 ARE. GAPDH primers were provided with the Millipore EZ-ChIP Chromatin Immunoprecipitation Kit. SEMA3C levels were quantitated using a ΔΔCt method, normalized first to input and then isotype control. Antibodies for immunoprecipitation: Androgen Receptor (Santa Cruz Biotechnology, Cat. No. sc-816), GATA-2 (Santa Cruz Biotechnology, Cat. No. sc-9008), and N-cadherin (isotype control, Santa Cruz Biotechnology, Cat. No. sc-7939).

### Luciferase assay

5×10^5^ LNCaP cells were transiently transfected in triplicate in 12-well format with 1.2 μg of either empty pGL3-Basic (Basic), pGL3-wild type ARE (wtARE), pGL3-mutated ARE (mutARE), or truncated pGL3-wtARE (wtARE-60bp and wtARE-120bp) reporter plasmids and 30 ng of renilla plasmid (phRL-SV40) kindly provided by the Mui lab (Immunity and Infection Research Centre, Vancouver Coastal Health Research Institute, Vancouver, British Columbia). 293T cells were co-transfected with or without AR or ARv7 overexpression plasmids or empty vector (pcDNA3.1). The following day the cells were treated with EtOH or R1881 in 5% CSS Opti-MEM. 24 hours later, cell extracts were harvested for luciferase assay using the Promega Dual-Luciferase Reporter Assay System (Cat. No. E1960) and read on a TECAN Infinite M200 PRO. For MDV3100 and 14449 dosing studies, 5×10^3^ LNCaP cells were seeded in quadruplicate in 96-well format, transfected with the wtARE construct (50 ng), and co-treated with R1881 (0.1 nM) and one of MDV3100 or VPC-14449 at the indicated concentrations (24 hrs) and read as described above. In all luciferase assays, firefly luciferase luminescence was normalized to renilla luciferase luminescence.

### Western blot

Whole cell extracts were prepared in 50 mM Tris-HCl, 150 mM NaCl, 1% NP40, 10 mM NaF, 10% Glycerol, supplemented with protease inhibitor cocktail (Roche, Cat. No. 04693116001) and quantitated using a BCA approach. 60 μg of protein, or 40 μl of conditioned media, was run on 10% acrylamide gels and transferred onto nitrocellulose membrane. Western blots were imaged on radiography film or by a LI-COR Odyssey system. Actin or vinculin served as loading controls. Primary antibodies: phospho-Akt (Ser473; Cell Signaling Technology, Cat. No. 4060S), pan-Akt, (Life Technologies, Cat. No. 44609G), androgen receptor (Santa Cruz Biotechnology, Cat. No. sc-816), SEMA3C (Santa Cruz Biotechnology, Cat. No. sc-27796), GATA2 (Santa Cruz Biotechnology, Cat. No. sc-9008), FOXA1 (Santa Cruz Biotechnology, Cat. No. sc-6553), POU2F1 (Santa Cruz Biotechnology, Cat. No.s sc-232, sc-8024; Cell Signaling Technology, Cat. No. 4428S), actin (Sigma-Aldrich, Cat. No. A2066), and vinculin (Sigma-Aldrich, Cat. No. V4505). Secondary antibodies: anti-rabbit alexa fluor 680 (Invitrogen, Cat. No. A21109), anti-mouse alexa fluor 680 (Invitrogen, Cat. No. A21058), anti-goat alexa fluor 680 (Invitrogen, Cat. No. A21084), anti-rabbit HRP (Dako, Cat. No. P0448), anti-mouse HRP (Dako, Cat. No. P0447), and anti-goat HRP (Dako, Cat. No. P0160).

### RNA knockdown

Cells were transfected with siRNA using Lipofectamine RNAiMAX (Invitrogen, Cat. No. 13778-075) for 48 hours at which time cells were either harvested or treated for an additional 24 hours (qPCR) or 48 hours (Western blot) with EtOH or R1881. Small interfering RNA for GATA2 (siGATA2) were purchased from Dharmacon (Cat. No. J009024-17-0005) and Ambion (Cat. No. 4392420, ID s5596); siFOXA1 was purchased from Ambion (Cat. No. 4392420, IDs s6687 and s6688); siPOU2F1 was purchased from Ambion (Cat. No. 4392420, ID s10849); siAR was purchased from Ambion (Cat. No. 4390824); negative control siRNA (siSCX) were purchased from Dharmacon (Cat. No. D001810-10-05) and Ambion (Cat. No. 4390843).

### Statistical analyses

Statistical analysis was performed using the Student's two-tailed t-test. Data are represented as mean ± SD unless otherwise stated. Data presented a representative of three biological replicates.

## SUPPLEMENTARY FIGURES


