# Supplementary material for: Androgen receptor transcriptionally regulates semaphorin 3C in a GATA2-dependent manner
Source: Oncotarget. 2016 Dec 25;8(6):9617–33. doi: 10.18632/oncotarget.14168 (PMC5354758; doi:10.18632/oncotarget.14168)
Supplement: Supplementary file 1 [file oncotarget-08-9617-s001.pdf]

Androgen receptor transcriptionally regulates semaphorin 3C in a GATA2-dependent manner

SUPPLEMENTARY FIGURES

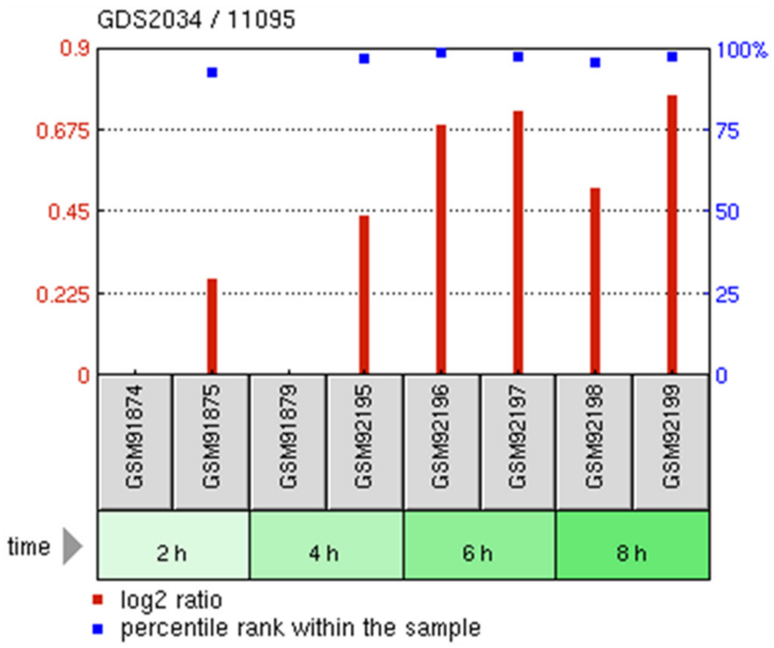

Supplementary Figure 1: Time-dependent increase in SEMA3C expression in response to R1881 in microarray. Data mining of GEO accession number GDS2034 show that the levels of SEMA3C mRNA increase over time in LNCaP treated with R1881. To identify targets of the AR, Hendriksen *et al* looked at expression profiles of LNCaP treated with R1881 for up to 8 hours. Samples: GSM91874: LNCaP_2h_R1881_rep1, GSM91875: LNCaP_2h_R1881_rep2, GSM91879: LNCaP_4h_R1881_rep1, GSM92195: LNCaP_4h_R1881_rep2, GSM92196: LNCaP_6h_R1881_rep1, GSM92197: LNCaP_6h_R1881_rep2, GSM92198: LNCaP_8h_R1881_rep1, GSM92199: LNCaP_8h_R1881_rep2.

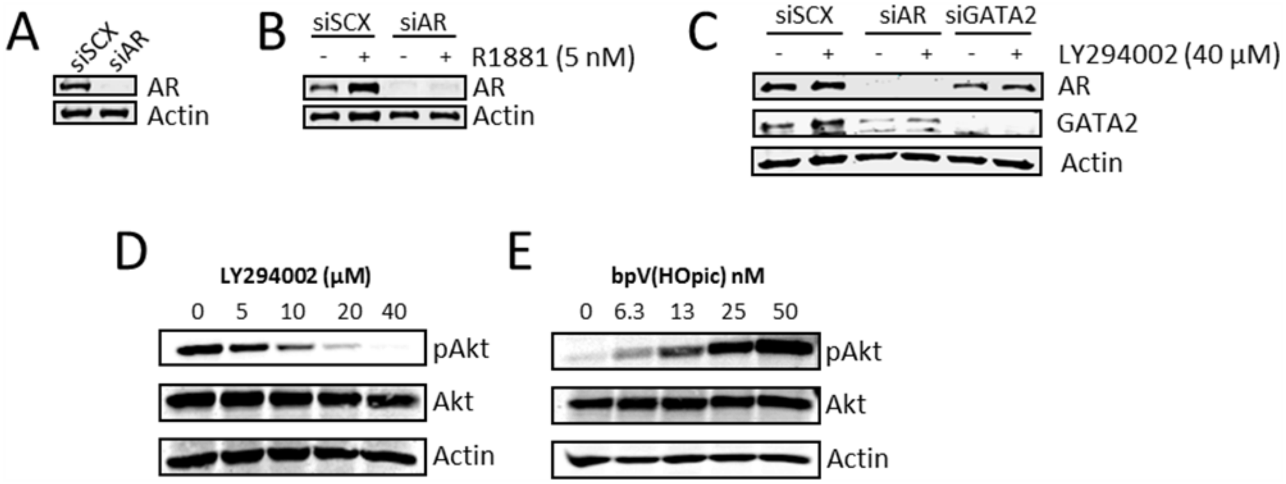

Supplementary Figure 2: Confirmational Western blots. **A** and **B.** Knockdown of AR for Figure 2I and 2J was confirmed by Western blot. **C.** Knockdown of AR and GATA2 for Figure 2L was confirmed by Western blot. **D** and **E.** Phospho-Akt was used to monitor the effects of LY294002 and bpV(HOpic) on PI3K activity. Total Akt and actin served as loading controls.

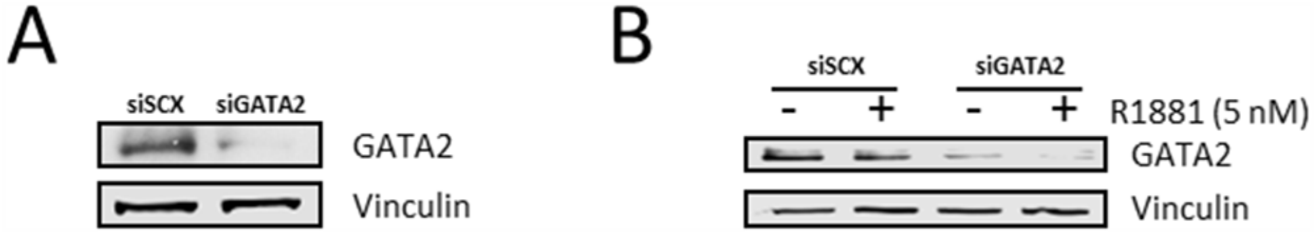

Supplementary Figure 3: Confirmation of knockdown of GATA2. A. Verification of knockdown of GATA2 for Figure 5A and 5B was confirmed by Western blot where vinculin served as a loading control. **B.** Knockdown of GATA2 for Figure 5E was confirmed by Western blot.

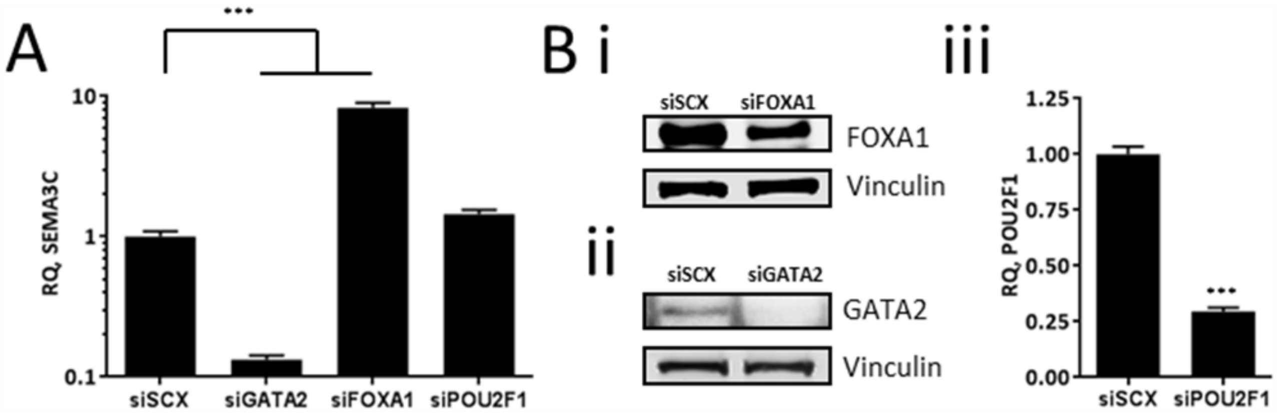

Supplementary Figure 4: GATA2 positively regulates SEMA3C expression and FOXA1 negatively regulates SEMA3C expression in C4-2 cells. A. C4-2 cells were separately knocked down with each of siGATA2, siFOXA1, and siPOU2F1, to confirm findings seen in LNCaP. Compared to C4-2 cells treated with scrambled siRNA (siSCX), C4-2 cells treated with siGATA2 had significantly diminished SEMA3C expression while C4-2 cells treated with siFOXA1 showed an upregulation of SEMA3C; C4-2 cells treated with siPOU2F1 showed a modest induction of SEMA3C. These results mirror those seen in LNCaP. **B.** Validation of knockdown was shown by Western blot analysis or qPCR. Data represent mean, ± SD; *** $p < 0.001$.
